# Amino Acid Changes at Arginine 204 of Troponin I Result in Increased Calcium Sensitivity of Force Development

**DOI:** 10.3389/fphys.2016.00520

**Published:** 2016-11-15

**Authors:** Susan Nguyen, Rylie Siu, Shannamar Dewey, Ziyou Cui, Aldrin V. Gomes

**Affiliations:** ^1^Department of Neurobiology, Physiology, and Behavior, University of California, Davis Davis, CA, USA; ^2^Department of Physiology and Membrane Biology, University of California, Davis Davis, CA, USA

**Keywords:** troponin I, familial hypertrophic cardiomyopathy, distal arthrogryposis, Ca^2+^ sensitivity, mammalian two-hybrid

## Abstract

Mutations in human cardiac troponin I (cTnI) have been associated with restrictive, dilated, and hypertrophic cardiomyopathies. The most commonly occurring residue on cTnI associated with familial hypertrophic cardiomyopathy (FHC) is arginine (R), which is also the most common residue at which multiple mutations occur. Two FHC mutations are known to occur at cTnI arginine 204, R204C and R204H, and both are associated with poor clinical prognosis. The R204H mutation has also been associated with restrictive cardiomyopathy (RCM). To characterize the effects of different mutations at the same residue (R204) on the physiological function of cTnI, six mutations at R204 (C, G, H, P, Q, W) were investigated in skinned fiber studies. Skinned fiber studies showed that all tested mutations at R204 caused significant increases in Ca^2+^ sensitivity of force development (ΔpCa_50_ = 0.22–0.35) when compared to wild-type (WT) cTnI. Investigation of the interactions between the cTnI mutants and WT cardiac troponin C (cTnC) or WT cardiac troponin T (cTnT) showed that all the mutations investigated, except R204G, affected either or both cTnI:cTnT and cTnI:cTnC interactions. The R204H mutation affected both cTnI:cTnT and cTnI:cTnC interactions while the R204C mutation affected only the cTnI:cTnC interaction. These results suggest that different mutations at the same site on cTnI could have varying effects on thin filament interactions. A mutation in fast skeletal TnI (R174Q, homologous to cTnI R204Q) also significantly increased Ca^2+^ sensitivity of force development (ΔpCa_50_ = 0.16). Our studies indicate that known cTnI mutations associated with poor prognosis (R204C and R204H) exhibit large increases in Ca^2+^ sensitivity of force development. Therefore, other R204 mutations that cause similar increases in Ca^2+^ sensitivity are also likely to have poor prognoses.

## Introduction

Familial hypertrophic cardiomyopathy (FHC) is a genetically heterogeneous autosomal dominant inherited heart disease of the myocardium that results in a high incidence of sudden cardiac death and has been linked to mutations in genes coding for at least 10 sarcomeric proteins. These mutations have been found in the genes for: α-myosin heavy chain (Geisterfer-Lowrance et al., 1990), cardiac myosin essential light chain, and cardiac myosin regulatory light chain (Poetter et al., 1996), α-tropomyosin (Thierfelder et al., 1994), cardiac troponin T (TnT) (Thierfelder et al., 1994), cardiac myosin binding protein C (Bonne et al., 1995; Watkins et al., 1995), cardiac troponin I (TnI) (Kimura et al., 1997), α-actin (Mogensen et al., 1999), troponin C (TnC) (Hoffmann et al., 2001), and titin (Satoh et al., 1999). FHC is the most commonly identified cause of sudden death in young adults. Symptoms of FHC vary between individuals, and most patients are asymptomatic (or only exhibit mild symptoms) before sudden cardiac death (O'Mahony and Elliott, 2014).

TnI is a basic protein that readily interacts with the acidic TnC and binds with another acidic protein, TnT, to form the Troponin (Tn) complex (Zot and Potter, 1987; Tobacman, 1996; Westfall and Metzger, 2001). TnI is also capable of binding actin and inhibiting Mg^2+^-activated actomyosin ATPase and is commonly referred to as the inhibitory subunit of Tn. The Tn complex and tropomyosin are critical regulatory components of the muscle contraction apparatus (Xu et al., 2010). Muscle contraction involves cyclic binding and release of cross-bridges (myosin heads of the thick filament interacting with and releasing actin of the thin filament) with energy derived from the hydrolysis of ATP.

Kimura et al. reported five missense mutations in TnI (R145G, R145Q, R162W, G203S, and K206Q) that are linked to FHC (Kimura et al., 1997). Since this initial report, several other TnI FHC mutants [examples include R21C, P82S, K183 deletion (ΔK183), I195M, D195N, S199N, R204C, R204H and an exon 8 deletion mutant encompassing the stop codon of the cTnI gene] have also been reported (Kokado et al., 2000; Morner et al., 2000; Niimura et al., 2002; Doolan et al., 2005). FHC mutations in TnI and other myofilament proteins have been previously shown by many groups to generally result in increased Ca^2+^-sensitivity of force development (Gomes and Potter, 2004). Since R204 had two known mutations (R204C and R204H), and arginine is the amino acid most commonly associated with FHC in TnI or TnT (Xu et al., 2010), it was hypothesized that any mutations at R204 would cause similar increases in Ca^2+^-sensitivity of force development. The R204 residue is an important amino acid to investigate since families with the cardiac TnI (cTnI) R204H mutation have a high incidence of sudden death (Doolan et al., 2005). Doolan et al. (2005), investigated R162G, R162P, L198P, R204H mutations and hypothesized that the FHC phenotype of missense mutations of arginine residues of TnI are due to disrupted functional interactions with TnC and TnT, which is tested in the present work using multiple residue replacements of R204.

The cTnI R204 residue is well conserved in different animals suggesting that this residue is evolutionarily important. A comparison of the primary amino acid sequence of cTnI and fast skeletal Troponin I (fsTnI) shows that R204 residue corresponds to the R174 residue of fsTnI. It is hypothesized that changes at the fsTnI R174 residue will show similar increases in Ca^2+^-sensitivity of force development as observed for the cTnI R204 mutants. Since a known missense mutation (R174Q) in fsTnI was shown to be associated with distal arthrogryposis type 2B (DA2B, also known as arthrogryposis multiplex congenita, distal, type 2B) (Westfall and Metzger, 2001), this mutation was also investigated in skeletal muscle to determine if this mutation showed similar *in vitro* physiological properties as the cTnI R204Q mutation. Distal arthrogryposis (DA) refers to a group of disorders characterized by multiple congenital contractures of the hands/wrists and feet/ankles, and affected individuals typically have a triangular shaped face, a small mouth, a prominent chin and positional foot deformities (calcaneovalgus and/or clubfoot).

Investigation of six cTnI R204 residues as well as the fsTnI R174Q mutation showed that all the mutations investigated increased the Ca^2+^ sensitivity of force development. The different effects of these mutants on the interaction between Tn subunits and the maximal force suggest that the change in Ca^2+^ sensitivity of force development may be a major determinant for the poor prognosis of patients with the cTnI R204C, R204H, and fsTnI R174Q mutations.

## Experimental procedures

### Mutation, expression, and purification of HcTnI and HcTnI mutants

The HcTnI FHC mutants were formed by overlapping PCR using HcTnI cDNA obtained from Dr. J.D. Potter (Zhang et al., 1995). Mutation, expression, and purification of cTnI and TnT mutants were carried out as previously described (Szczesna et al., 2000; Gomes et al., 2005b). The PCR products obtained were digested with NcoI and BamH1 and then ligated to pET-3d vector. The sequence of the TnI mutants was verified by sequencing prior to expression and purification. HcTnI and HcTnI R204 mutants were purified via conventional methods. Briefly, crude bacterial supernatants were purified by column chromatography on an S-Sepharose column at 4°C and eluted with a linear KCl gradient of 0–0.5 M in a Tris-HCl buffer containing 6M urea. Semi-pure HcTnI and HcTnI mutantswere dialyzed against a solution containing 50 mM Tris-HCl, pH 7.5, 1 M KCl, 1 M urea, 1 mM DTT, and 2 mM CaCl_2_ and loaded onto an affinity column having covalently bound HcTnC. Pure HcTnI and HcTnImutants were eluted with a gradient of 0–3 mM EDTA and 1–6 M urea. The purity of the TnI proteins was determined by SDS-PAGE as previously described (Gilda et al., 2016).

### Mutation and expression of wild-type and mutant fast skeletal TnI

The cDNA encoding rabbit fsTnI was previously cloned by reverse transcriptase-polymerase chain reaction using a template of total RNA from rabbit skeletal muscle and oligonucleotide primers specific for the 5′ and 3′ regions of the respective coding sequences (Sheng et al., 1992). Additionally, the fsTnI R174Q mutant was made using a sequential overlapping polymerase chain reaction-based method as previously described (Gomes et al., 2004, 2005a). Clones were sequenced to verify the correct sequences prior to expression and purification of the respective proteins.

### Protein purification of rabbit fast skeletal TnT, TnI, and TnC

The purification of fsTnI was similar to previously described (Pan and Potter, 1992) with the exception that the final purification step was with TnC-agarose affinity chromatography. Briefly, after S-Sepharose cation exchange chromatography in the presence of 6M, fsTnI was eluted with a continuous ionic strength gradient of 0 –0.3 M NaCl. The fsTnI containing fractions were pooled and dialyzed in steps (to remove urea) with 6M urea, 4 M urea, 2 M urea in TnC affinity buffer to TnC affinity buffer with no urea (50 mM Tris, pH 7.5 containing 2 mM CaCl_2_, 0.5M NaCl, and 1 mM DTT). A column with cardiac TnC immobilized onto CNBr-activated Sepharose-4B (2 mg TnC/ml agarose) was prepared as described by Pharmacia LKB Biotechnology. The dialyzed TnI was loaded onto the TnC-agarose column equilibrated with TnC affinity buffer. The bound TnI was eluted with TnC elution buffer containing 6 M urea and 3 mM EDTA (50 mM Tris, 0.5 mM NaCl, 1 mM DTT, 3 mM EDTA, 6 M urea, pH 7.5). The eluates were run on SDS gels and purified TnI pooled.

### Formation of the troponin complex

Formation of the human cardiac troponin and rabbit skeletal troponin complexes containing recombinant TnT, TnC, and TnI was carried out as recently described by Szczesna et al. (2000), (Szczesna et al., 2000; Gomes et al., 2005b). Proper stoichiometry was verified by SDS-PAGE (Gilda et al., 2016). Although not done routinely, gel filtration of these formed Tn complexes showed that this reconstitution method resulted in a single species.

### Preparation of porcine skinned cardiac muscle bundles

Cardiac skinned muscle fibers were prepared following a common laboratory procedure published by Zhang et al. (1995) (Zhang et al., 1995; Gomes et al., 2005a,b). Freshly isolated porcine hearts were incubated in an O_2_-saturated solution containing 140 mM NaCl, 4 mM KCl, 1.8 mM CaCl_2_, 1 mM MgCl_2_, 1.8 mM NaHPO_4_, 5.5 mM glucose, and 5 mM HEPES, pH 7.4. Cardiac muscle bundles were dissected from the left ventricle of the porcine hearts and were chemically skinned by incubating with 50% glycerol and 1% Triton X-100 in the relaxing solution (pCa 8.0) containing 10^−8^ M Ca^2+^, 5 mM Mg^2+^, 7 mM EGTA, 20 mM MgATP, 20 mM creatine phosphate, and 15 U/mL creatine phosphokinase, pH 7.0, at an ionic strength of 150 mM at 4°C for 24 h. These skinned muscle preparations were dissected into small bundles (1–2 cm in length, 2–3 mm in diameter) and were stored at –20°C in the same solution without triton X-100 before use.

### Steady-state force and Ca^2+^-sensitivity of force development of Tn complexes containing HcTnI mutants

The skinned fiber preparation was mounted with stainless steel clips on a force transducer and was immersed in the contracting solution to measure initial force before treatment. The contraction solution (pCa 4) had the same composition as the relaxation buffer except for the increased Ca^2+^ concentration (10^−4^ M). To determine the Ca^2+^ dependence of force development, the contraction of the skinned fibers was tested in solutions of intermediate concentrations of Ca^2+^. The Ca^2+^ dependence of force was determined before and after performing the displacement and reconstitution protocols that are described below. To remove the endogenous Tn complex, the TnT displacement method was used (Hatakenaka and Ohtsuki, 1992; Szczesna et al., 2000; Gomes et al., 2005a). The cardiac fiber was incubated with HCTnT (0.8 mg/mL) for a total of 2.5 h at room temperature with an intermediate buffer change containing fresh HCTnT (0.8 mg/mL). After displacement of the endogenous complex by HCTnT, the level of unregulated force development was observed by measuring the level of force reached by skinned fibers in both the pCa 8 solution and the pCa 4 solutions. Restoration of the Ca^2+^ regulation of force development was performed using troponin I:troponin C complexes (30 μm) in pCa 8 solution for ~1.5 h at room temperature. To determine the Ca^2+^ dependence of force development, the contraction of skinned fibers was tested in solutions with intermediate concentrations of Ca^2+^ (from pCa 8 to pCa 4). The Ca^2+^ dependence was determined before and after treatment of the skinned fibers with displacement and reconstitution solutions. The Ca^2+^ dependence data were analyzed using the Hill equation (Sigmaplot, Jandel Scientific): relative force (%) = [Ca^2+^]^n^/([Ca^2+^]^n^+[pCa_50_]^n^), where pCa50 is the pCa of a solution in which 50% of the change is produced, and n is the Hill coefficient.

### Steady-state force and the Ca^2+^-sensitivity of force development of Tn complexes containing wild-type and fsTnIR174Q

Skeletal muscle fiber bundles from rabbit psoas muscle were mounted on a force transducer and treated with the pCa 8 relaxing solution containing 1% Triton X-100 for ~ 1 h. The composition of the pCa 8 solution was 10^−8^ M [Ca^2+^], 1 mM [Mg^2+^], 7 mM EGTA, 5 mM [MgATp^2+^], 20 mM imidazole, pH 7.0, 20 mM creatinine phosphate, and 15 units/ml creatinine phosphokinase, ionic strength = 150 mM. To determine the Ca^2+^ sensitivity of force development, the fibers were gradually exposed to the solutions of increasing Ca^2+^ concentrations, from pCa 8 to pCa 4. To displace the endogenous Tn complex from the fibers they were incubated in a solution containing 250 mM KCI, 20 mM MOPS, pH 6.2, 5 mM MgCl_2_, 5 mM EGTA, 0.5 mM dithiothreitol, and 1.6–1.8 mg/ml fsTnT, for 1 h at room temperature. A fresh fsTnT protein was applied to the fibers for another 1 h incubation. This was to increase the efficiency of the endogenous Tn displacement from the fibers. Displaced fibers were then washed with the same solution without the protein (10 min at room temperature) and tested for Ca^2+^-unregulated force that developed due to the absence of the endogenous TnI and TnC. The Ca^2+^ regulation of steady-state force was restored with a preformed fsTnI·fsTnC complex. The reconstitution with the fsTnI:fsTnC complex (25 μM) was performed in the pCa 8 solution for ~1.5 h at room temperature, or long enough for the force to reach a stable level. Control fibers were run in parallel and treated with the same solutions minus the proteins. The final Ca^2+^-sensitivity of force development was determined after fsTnI·fsTnC reconstitution and the data were analyzed with the Hill equation.

### Mammalian two-hybrid studies

Protein:protein interactions were measured using the Checkmate Mammalian Two-Hybrid System (Promega) as previously described (Gilda et al., 2016). cTnI WT and cTnI deletion mutants as well as WT cTnT, and cTnC were subcloned into pACT and pBIND vectors. CV1 cells were transfected with pACT and pBind DNA constructs as well as the pG5luc Vector. TransIT-LT1 transfection reagent (Mirus) was used for all experiments and cells were incubated for 36–48 h following transfection with no media change. Cells were lysed and analyzed using the dual luciferase reporter assay (Promega). To verify the experiments were working correctly, control experiments using various combinations of empty vectors, cTnI, cTnC, or cTnT subcloned vectors were also carried out as previously described (Gilda et al., 2016). To control for potential differential protein expression in the two-hybrid assay, protein levels of the wild-type, and TnI mutants (cloned into the mammalian two hybrid plasmids) in CV1 cells were initially checked by Western blotting to determine if any of the mutants investigated showed significantly different expression levels. All TnI mutants were found to show similar expression levels after 48 h.

### Statistical methods

All data are presented as mean ± S.D. Comparisons of mammalian-two hybrid data and maximal force data were carried out using one way ANOVA. Unpaired Student's *t*-test was used to determine the significance of differences in ΔpCa (changes in half-maximal activating pCa in different skinned fibers). Values of *P* < 0.05 were considered statistically significant.

## Results

**Figure 1:**
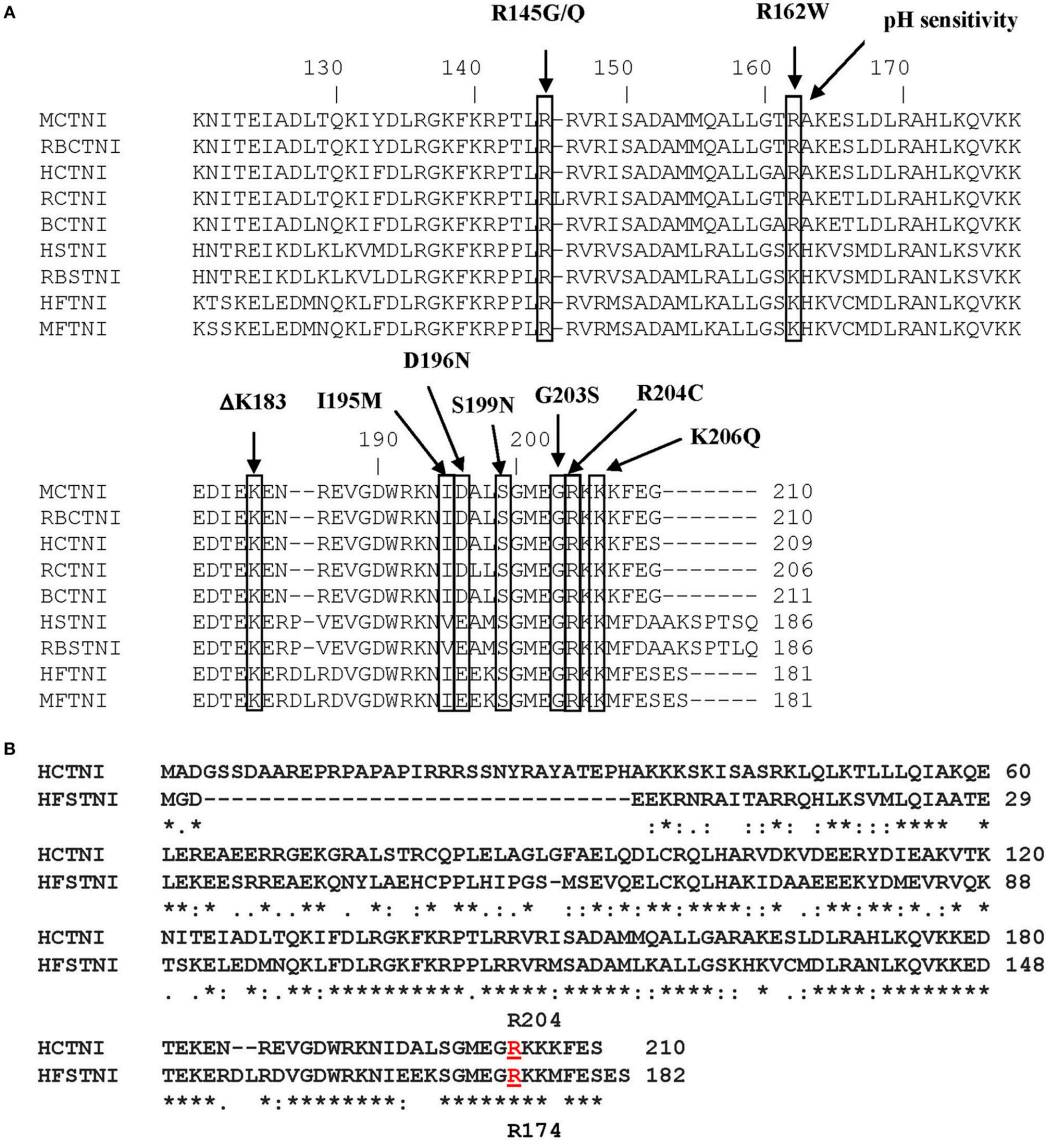
**Primary structure of Human Cardiac Troponin I. (A)** Alignment of the primary sequences of cardiac and fast skeletal Troponin I from different species. Some of the cTnI FHC mutations that occur within this region are indicated. The location of the R204 mutation is indicated. M, mouse; RB, rabbit; H, human; R, rat; B, bovine. **(B)** Alignment of the primary sequences of Human cardiac Troponin I (HCTNI) and Human fast skeletal Troponin I (HFSTNI). Human cardiac Troponin I (Swiss-Prot accession number: P19429). Human fast skeletal Troponin I (Swiss-Prot accession number: P48788). ^*^Indicates identical amino acid.: indicates homology.

Figure 1A shows the primary structure of the C-terminal region of cTnI with the cTnI FHC mutations that occur within this region indicated. Figure 1B shows the primary sequence alignment for HcTnI and human fsTnI (HfsTnI) indicating that the position of the R204 mutation in HcTnI corresponds to R174 in HfsTnI.

### Force development and the Ca^2+^ dependence of force development for cTnI mutations

To determine the effect of these mutations (R204C and R204H), as well as other potential cTnI mutations, R204G, R204P, R204Q, and R204W, cardiac skinned fiber calcium-force measurements were carried out. We employed a well-established method in our lab (Szczesna et al., 2000; Gomes et al., 2002; Lang et al., 2002) to displace the endogenous Tn complex from skinned porcine cardiac muscle preparations. After determining the level of unregulated force in skinned fibers, they were incubated with either wild-type or mutant HcTnI·HcTnC complexes in low calcium buffer (pCa 8). This allowed us to determine whether the TnI proteins could fully inhibit Ca^2+^ unregulated force established after treatment with HcTnT and also to determine if the proteins were able to fully reconstitute the skinned fibers by forming a functional Tn complex. Wild-type HcTnI·HcTnC complex resulted in complete inhibition of Ca^2+^ unregulated force.

**Figure 2:**
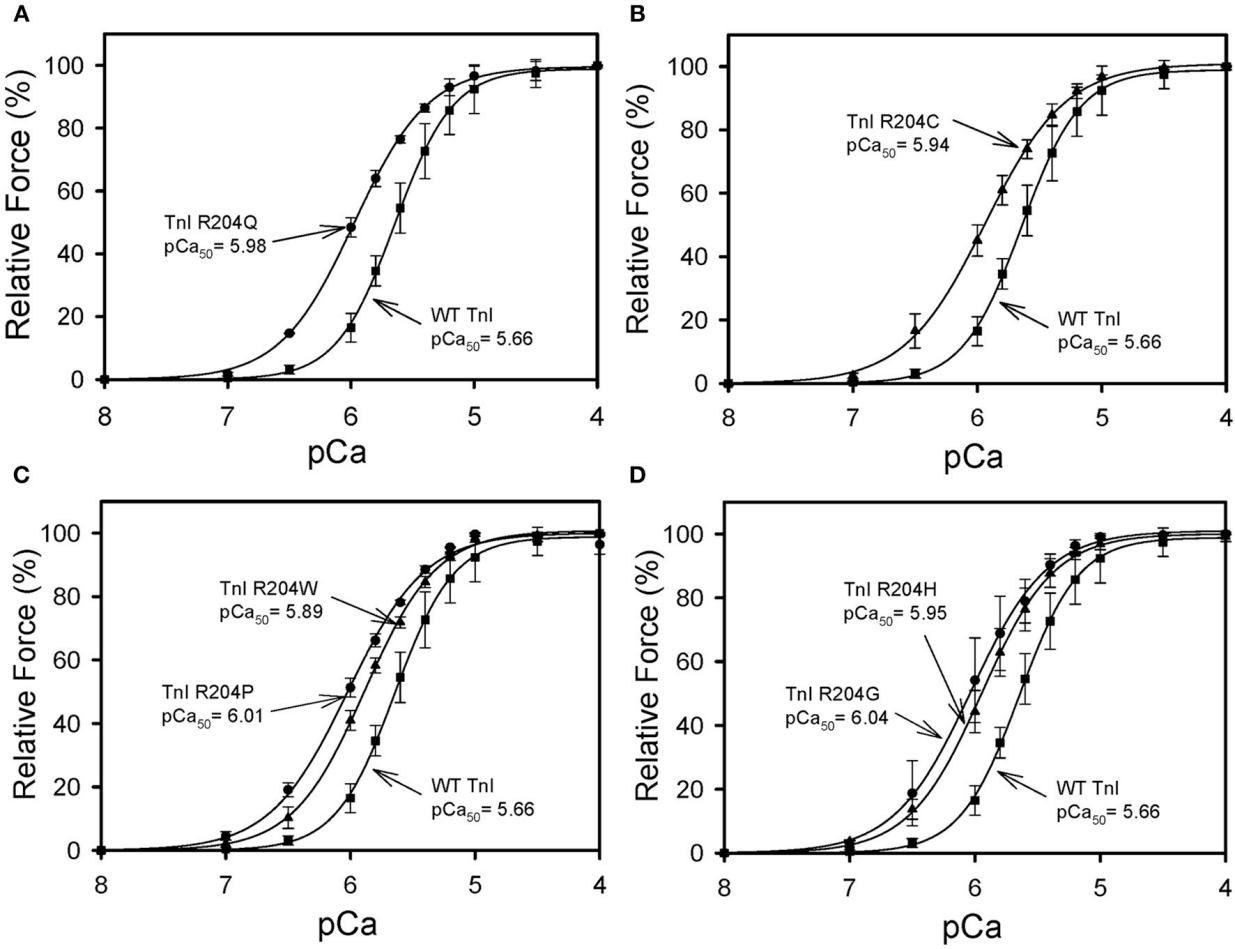
**Effect of mutations in Troponin I at R204 on the calcium sensitivity of force development**. Each skinned muscle preparation was treated with HcTnT to displace the endogenous Tn complex and subsequently reconstituted with either HcTnI·HcTnC, or HcTnI R204 mutant. **(A)** Comparison of HcTnI·HcTnC and HcTnI R204Q·HCTnC. **(B)** Comparison of HcTnI·HcTnC and HcTnI R204C·HCTnC. **(C)** Comparison of HcTnI·HcTnC with HcTnI R204W·HcTnC or HcTnI R204P·HcTnC. **(D)** Comparison of HcTnI·HcTnC with HcTnI R204H·HcTnC or HcTnI R 204G·HcTnC. The Ca^2+^ dependence of force was measured in each preparation after reconstituting troponin. Each point is the average of 3 experiments and represents the mean ± S.D. ^*^p < 0.05.

**Figure 3:**
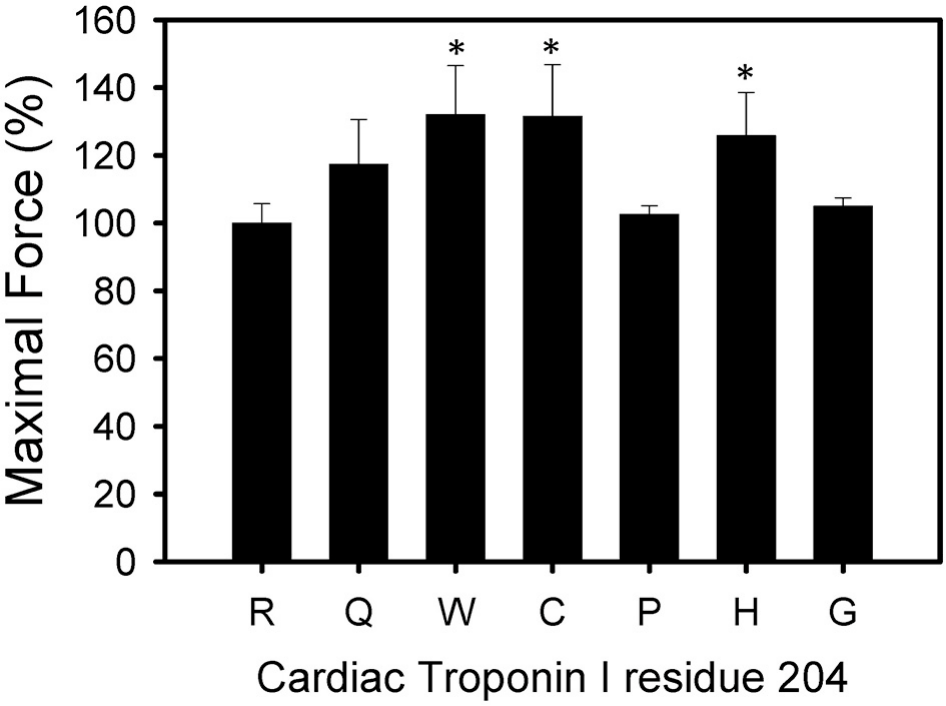
**Effect of mutations in Troponin I at R204 on Maximal force**. Each bar is the average of 3 experiments and represents the mean ± S.D. Forces were normalized to the maximum force developed by each fiber after TnT treatment and HcTnI·HcTnC reconstitution. ^*^P < 0.05 (*n* = 3).

All six mutations showed significant increases in calcium sensitivity of force development ranging from ΔpCa_50_ 0.22 (R204W) to 0.37 (R204G) (Figure 2, Table 1). The mutations associated with FHC, R204C, and R204H, had ΔpCa50 values of 0.27 and 0.28 respectively (Figure 2, Table 1). Recovered force is equivalent to the level of force developed in fibers after reconstituting the fibers with the appropriate Tn complex. The maximal force (force at pCa 4.0) obtained from the skinned fibers was increased for cTnI R204W, R204C, and R204H mutations relative to wild-type cTnI (Figure 3). It is interesting that both known FHC mutations, R204C and R204H, increase maximal force.

**Table 1:** **Effect of wild-type Human cTnI and mutants on the Ca^**2+**^ sensitivity of force development (pCa_**50**_) and the Hill coefficient (n_**H**_) in skinned porcine cardiac muscle fibers**.

**Human cTnI Mutant**	**pCa_50_**	**Difference from Wild-type (ΔpCa_50_)**	**Hill coefficient (n_H_)**	**Number of experiments (n)**
Wild-type (204R)	5.66 ± 0.07	**-**	1.85 ± 0.23	3
204H	5.95 ± 0.08^*^	0.28	1.52 ± 0.08^*^	3
204G	6.04 ± 0.17^*^	0.37	1.51 ± 0.09^*^	3
204C	5.94 ± 0.07^*^	0.27	1.35 ± 0.07^*^	3
204W	5.89 ± 0.03^*^	0.22	1.50 ± 0.03^*^	3
204Q	5.98 ± 0.04^*^	0.31	1.46 ± 0.02^*^	3
204P	6.01 ± 0.01^*^	0.35	1.40 ± 0.06^*^	3

*The pCa_50_ and n_H_ values are the average of 3 independent fiber experiments, and the errors are the standard deviation (S.D.) values.*

* *Indicates that the pCa_50_ values and Hill coefficient for the respective TnI mutants are significantly different from wild-type HcTnI (p < 0.05)*.

#### Mammalian two-hybrid

To determine the effect of the R204 mutations on cTnI: cTnT and cTnI: cTnC interactions, the mammalian two-hybrid luciferase assay was utilized. In the CheckMate mammalian two-hybrid system utilized, the pACT vector contains the herpes simplex virus VP16 activation domain upstream of the cloning region, while the pBIND vector contains the yeast GAL4 DNA-binding domain upstream of the cloning region. The pBIND Vector also expresses *Renilla reniformis* luciferase under the control of the SV40 promoter, which allows the normalization for differences in transfection efficiency. The troponin subunits were cloned into pBIND and pACT Vectors to generate fusion proteins with the DNA-binding domain of GAL4 and the activation domain of VP16, respectively. Association of the DNA-binding domain and the transcriptional activation domain results in transcriptional activation of the firefly luciferase reporter gene and an increase in firefly luciferase expression when compared to the negative controls. Mutations in interacting proteins that disrupt or significantly reduce the interactions between the two proteins being investigated would result in reduced association between the DNA-binding domain and the transcriptional activation domain resulting in less firefly luciferase expression.

**Figure 4:**
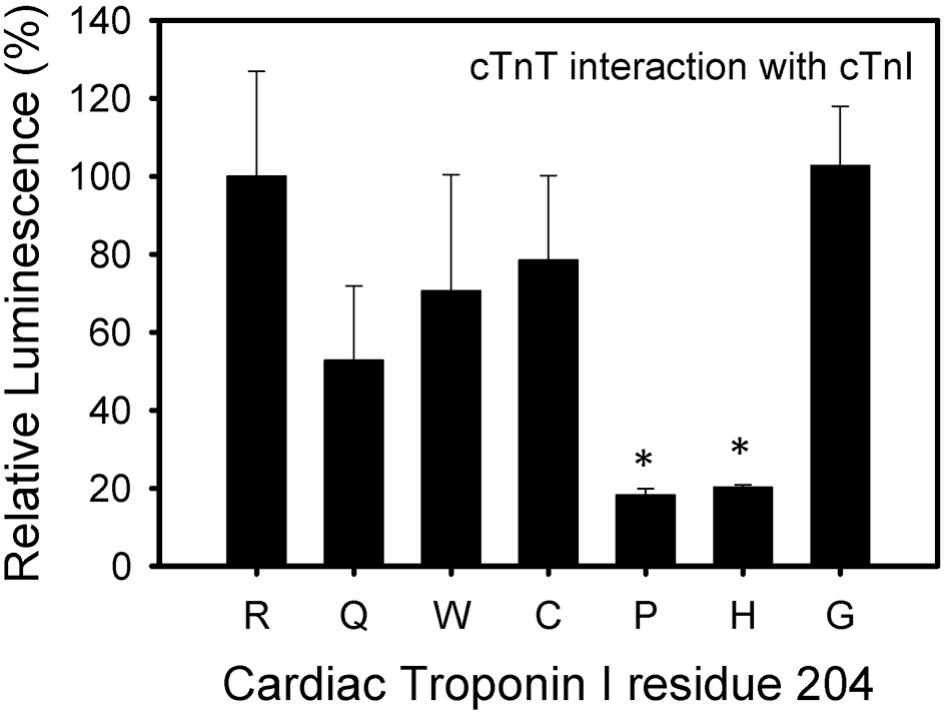
**Effect of mutations in Troponin I at R204 on its interaction with Troponin T**. Mammalian two-hybrid was utilized to determine disruptions in the interactions between different cTnI's and wild-type cTnT. ^*^P < 0.05 (*n* = 4–5).

**Figure 5:**
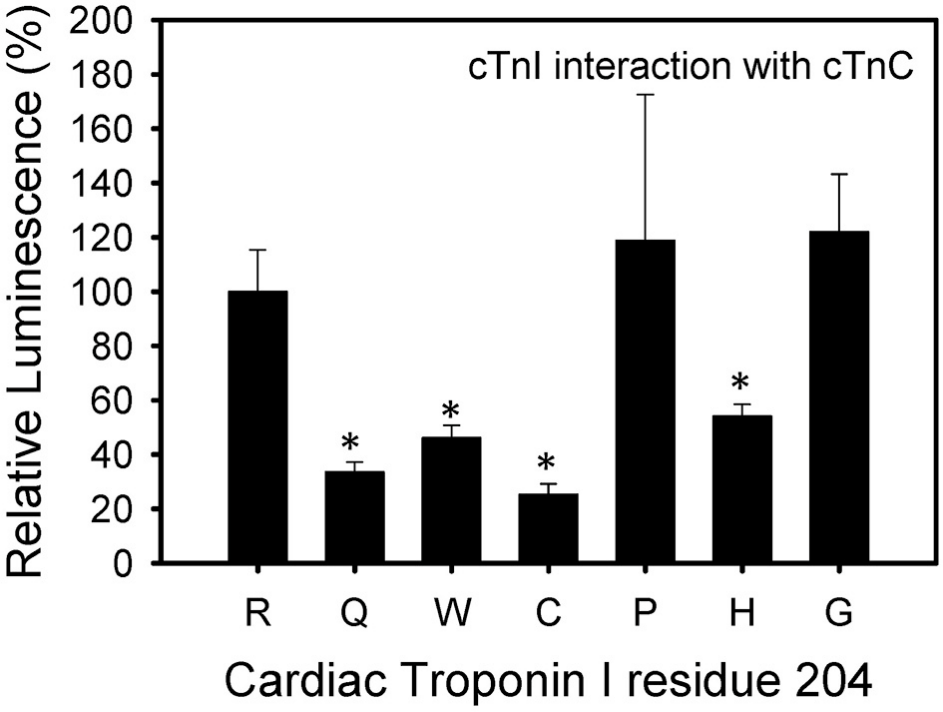
**Effect of mutations in Troponin I at R204 on its interaction with Troponin C**. Mammalian two-hybrid was utilized to determine disruptions in the interactions between different cTnI's and wild-type cTnC. ^*^P < 0.05 (*n* = 4–5).

The cTnI R204P and R204H mutations showed the weakest interactions with cTnT when compared to wild-type cTnI (Figure 4). The other mutations, including R204C, showed no significant impairment when compared to wild-type cTnI. These results suggest differences in the interactions between R204C and R204H with cTnT. Four of the six mutations investigated (R204Q, R204W, R204C, R204H) showed weaker interactions with cTnC (Figure 5). The cTnI R204P mutation which showed weaker interaction with cTnT, interacted with cTnC similarly to wild-type cTnI (Figure 5). The cTnI R204G mutant also did not show any significant impairment in cTnC binding when compared to wild-type cTnI. The disruption in binding between cTnI and its binding partners may be due to conformation changes in cTnI caused by the mutations.

### Force development and the Ca^2+^ dependence of force development for fsTnI

Rabbit fsTnI was studied in place of HfsTnI because it is the best characterized fast skeletal muscle system and the C-terminal half of rabbit fsTnI shows 100% homology with HfsTnI. The homology between rabbit fsTnI and HfsTnI is 98.9%. Under the conditions utilized, essentially all of the endogenous Tn complex was displaced following treatment with fsTnT. The amount of force development in the presence of very low concentration of Ca^2+^ (pCa 8.0) after fsTnT displacement is a measure of the extent of displacement of endogenous fsTnI (referred to as the Ca^2+^ unregulated force). In the absence of fsTnI, fibers were unable to relax, as the inhibitory activity of cTnI is deficient. This measurement of Ca^2+^ unregulated force was utilized to ensure that all the skinned fibers are displaced to the same extent. All the fibers selected for these studies had a Ca^2+^ unregulated force of >95%.

**Figure 6:**
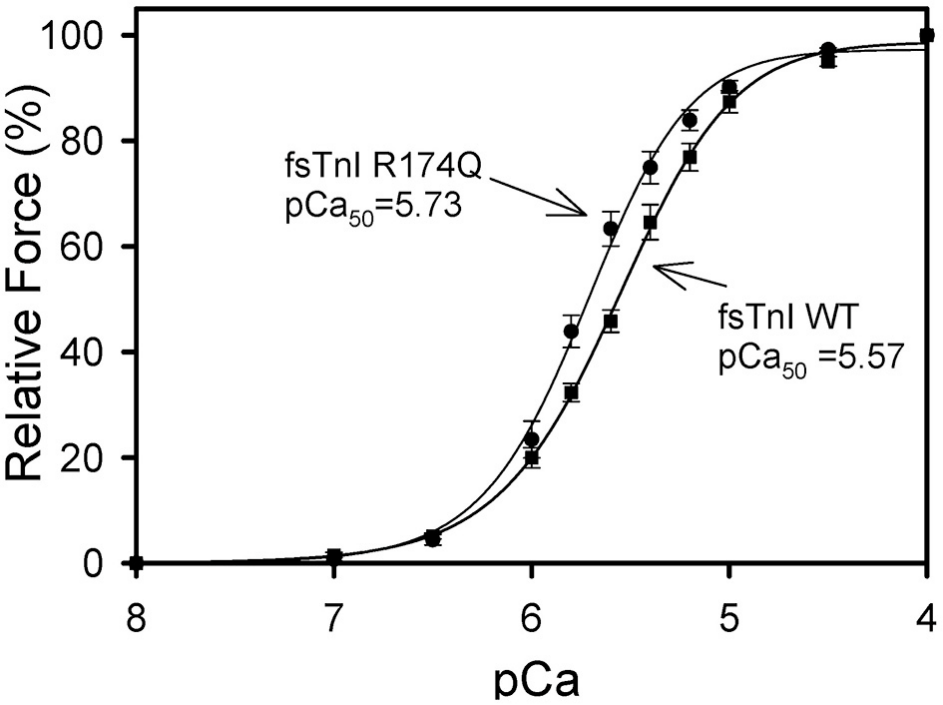
**Effect of Wild-type Fast Skeletal TnI and TnI R174Q on Ca^**2+**^-sensitivity of force development in skinned rabbit muscle fibers**. After TnT treatment the fibers were reconstituted with TnI·TnC complex. Comparison of rabbit fibers after they were reconstituted with wild-type fsTnI or fsTnI R174Q in the presence of fsTnC. The Ca^2+^ dependence of force was measured in each preparation after reconstituting whole Tn. Each point is the average of 3–5 experiments and represents the mean ±*S.D*.

**Figure 7:**
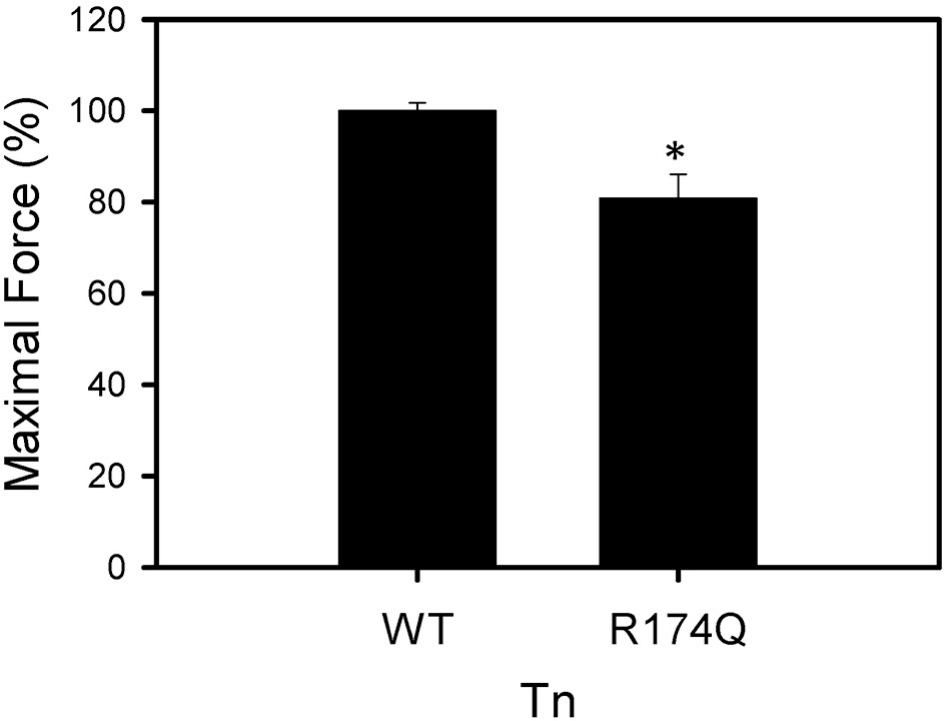
**Effect of Wild-type Fast Skeletal TnI and TnI R174Q on force development in skinned muscle fibers**. Maximal force development of fibers after fsTnT displacement and fsTnI·fsTnC or fsTnI R174Q·fsTnC reconstitution. Each bar is the average of 3–5 experiments and represents the mean ± *S.D*. ^*^P < 0.05.

After displacement and reconstitution, the maximal force obtained for each fiber was measured. This force was measured relative to the initial force of the skinned fibers before TnT displacement. An R174Q fsTnI mutation (associated with DA) increased the Ca^2+^ sensitivity of force development (Figure 6, Table 2). The results suggest that mutations associated with significantly increased Ca^2+^ sensitivity of force development may be associated with HCM in cardiac tissue or DA in skeletal muscle. Unlike some of the cardiac R204 mutations which showed increased maximal force, the fsTnI R174Q mutant showed decreased maximal force relative to wild-type fsTnI (Figure 7).

**Table 2:** **Effect of wild-type Rabbit Fast Skeletal TnI and TnI R174Q on the Ca^**2+**^-sensitivity of force development (pCa_**50**_) and the Hill coefficient (***n***_***H***_) in skinned fast skeletal muscle fibers**.

**Fast skeletal TnI**	**pCa_50_**	**Difference from Wild-type (ΔpCa_50_)**	**Hill Coefficient (*n*_*H*_)**	**Number of experiments (n)**
Wild-type fsTnI	5.57 ± 0.01	-	1.64 ± 0.13	3
fsTnI R174Q	5.73 ± 0.03^*^	0.16	1.65 ± 0.10	5

* *Indicates that the pCa_50_ values for the respective TnI mutants are significantly different from wild-type HcTnI (P < 0.05). The pCa_50_ and nH values are the average of 3-5 independent fiber experiments, and the errors are the standard deviation (S.D.) values. The average wild-type TnI displacement was 97.6 ± 2.4%*.

## Discussion

Arginine residues in cTnI are associated with multiple mutations resulting in potentially different clinical phenotypes (Xu et al., 2010). Since two mutations (R204C and R204H) at residue 204 of cTnI are associated with FHC and most mutations associated with FHC show increased Ca^2+^-sensitivity of force development (Xu et al., 2010), we hypothesized that any mutations at cTnI R204 would cause significant increases in Ca^2+^-sensitivity of force development. To characterize the effects that different arginine mutations at the same residue would have on the physiological function of cTnI, six mutations at R204 (C, G, H, P, Q, W) were investigated in skinned fiber studies. All of the cTnI R204 mutations investigated displayed increased Ca^2+^ sensitivity of force development when compared to WT cTnI (ΔpCa_50_ = 0.22–0.37), similar to what is observed for most FHC mutations. These results suggest that the R204 residue is important in cTnI function and that increased Ca^2+^ sensitivity may be a major factor in HCM. The increase in myofilament sensitivity to Ca^2+^ is important since reduction in the Ca^2+^ sensitivity was found to prevent the development of HCM (Alves et al., 2014). Others have suggested that increased Ca^2+^-sensitivity is associated with HCM (Wei and Jin, 2015).

Certain regions of cTnI seem to be more important functionally than other regions (Mogensen et al., 2015; Wei and Jin, 2015; Meyer and Chase, 2016; Sheng and Jin, 2016). It may be that any or most amino acid changes at certain TnI residues that occur in regions functionally important for regulating Ca^2+^-sensitivity would all be associated with increased Ca^2+^-sensitivity of force development. Transgenic mice expressing 9–17% of a C-terminal truncated human cardiac TnI (residues 194–210 deleted, corresponding to residues 166–182 of fsTnI), developed a phenotype of stunned myocardium (Murphy et al., 2000). These results suggest that the 194–210 region of cTnI is important for cTnI function. The FHC mutations R204C and R204H both showed similar increases in Ca^2+^ sensitivity (ΔpCa50 values of 0.28 and 0.29 respectively). These two mutations also showed increased maximal force compared to WT cTnI. R204W also showed increased maximal force. The R204H mutation was found to be associated with HCM in an Australian family and is associated with a poor prognosis (Doolan et al., 2005).

To determine how R204 mutations might disrupt protein-protein interactions, a mammalian two-hybrid luciferase assay was used. Mutation of arginine to proline resulted in a significant reduction in functional interactions with both cTnC and cTnT. The results suggest that binding of cTnI mutants (except R204G) to its binding partners is disrupted pending measurement of actual affinity constants. Interestingly, while the R204C mutation weakened the interaction of TnI with TnC, it did not significantly affect the interaction of TnI with TnT. However, the cTnI R204H mutation disrupted the interaction between cTnI and cTnT as well as between cTnI and cTnC, which is consistent with a previous report using a similar mammalian two hybrid assay (Doolan et al., 2005). Doolan et al. found evidence for altered interactions between TnI and either TnT, TnC, or both, for cTnI R162G, R162P, L194P, and R204H mutations. The previous finding that the cTnI R162G mutation reduced cTnI and cTnT interaction while another mutation at the same cTnI residue, R162P, did not affect cTnI-cTnT interaction (Doolan et al., 2005) is consistent with our results which suggest that not all cTnI HCM mutations affect cTnI-cTnT mutations. Using the data obtained from the four mutations they investigated, Doolan et al. concluded that altered interactions in the Tn complex “increased severity of the disease.” The replacement of cTnI arginine residue 204, a relatively large and charged amino acid, by glycine, the smallest amino acid, resulted in the largest increase in Ca^2+^-sensitivity of force development. The severity of the disease is likely to be more complicated due to altered interactions in the Tn complex since our R204G results suggest increased Ca^2+^-sensitivity of force development independent of any change in interactions with its Tn binding partners.

Replacement of arginine with proline also resulted in a large increase in Ca^2+^ sensitivity of force development, which was significantly larger than the increase in Ca^2+^ sensitivity of force development observed for the R204W mutation (*p* < 0.05). This difference in Ca^2+^ sensitivity changes may be due to the size of the tryptophan residue, which is the largest amino acid and closer to the size of arginine than proline or glycine (Morris et al., 1992). Proline is also typically considered a structural disruptor of the protein secondary structure and its side chains are conformationally rigid, unlike glycine which can easily adopt many main chain conformations.

To extend the hypothesis that any mutation at cTnI R204 would cause significant increases in Ca^2+^ sensitivity of force development, we hypothesized that mutations at fsTnI R174, which occurs at an equivalent location to the cTnI R204 residue, would also cause significant increases in Ca^2+^-sensitivity of force development. We investigated the R174Q missense mutation associated with DA. The WT rabbit fsTnI consists of 182 residues, and the C-terminus region of fsTnI, which is affected by the R174Q mutation, is known to interact with TnC and actin. The fsTnI R174Q DA mutant showed increased Ca^2+^-sensitivity of force development, similar to what was observed for the cTnI R204Q mutation. Hence, the location of the R174Q DA mutation is likely to be physiologically important for the function of TnI. The R174Q was previously investigated by another group that showed a greater increase in Ca^2+^-sensitivity of force development (ΔpCa_50_ = 0.36) and no change in maximal force (Robinson et al., 2007). The differences from what we observed may be due to species of cTnI utilized, as Robinson et al. utilized human fsTnI in muscle fibers in rabbit skeletal muscle fibers while we utilized rabbit fsTnI in rabbit skeletal muscle fibers (Robinson et al., 2007). However, in both cases the increase in Ca^2+^-sensitivity of force development relative to WT fsTnI was significant.

DA occurs in about 1 in 3000 children, and other fsTnI mutations have been found to be associated with DA2B (fsTnI R156ter, K175del, and K176del mutations) (Kimber et al., 2012). Patients with DA2B have contractures which are present at birth, suggesting impaired relaxation of muscle fibers. A likely possibility is that the higher Ca^2+^-sensitivity of force development in fibers containing DA2B mutations affects muscle contracture. These results, as well as two other reports that showed increased Ca^2+^-sensitivity with DA2B mutations (Robinson et al., 2007; Mokbel et al., 2013), all suggest that joint and muscle contractures may be caused by prolonged muscle hypercontraction due to increased myofibrillar calcium sensitivity and that treatments that reduce skeletal muscle Ca^2+^ sensitivity may be beneficial to patients with DA2B.

Overall, these results suggest that different amino acids at the same site on cTnI could affect thin filament interactions differentially. While significant impairment in the interactions of cTnI with TnT or TnC may be enough to cause significant changes in Ca^2+^-sensitivity of force development, impairment in these interactions is not a requirement for altering the Ca^2+^-sensitivity as our study showed that R204G had the largest increase in Ca^2+^-sensitivity yet demonstrated maximal tension and cTnI:TnT/TnC interactions comparable to wild-type levels. While R204G did not show any altered interactions between cTnI and TnC or TnT it is possible that altered interactions between TnI R204G and actin may occur. Unfortunately, we were unable to get cTnI:actin two hybrid studies to work well. It may also be possible that the R204 mutations are affecting thin and thick filament interactions by disturbing tropomyosin displacement. If the large increase in Ca^2+^-sensitivity of force development observed with these mutations is associated with the poor prognosis, then many other R204 mutations that may be discovered in the population are likely to have poor prognoses.

## Ethics statement

This study was exempt from this requirement because the tissues were obtained from the University of California, Davis Veterinary School Slaughterhouse. The Veterinary School Slaughterhouse routinely sacrifices animals for teaching purposes and sells the meat from these animals to the public. A qualified researcher can pay a small fee ($10) and get heart tissue or some skeletal muscle for research from these animals that have already been sacrificed.

## Author contributions

AG: design of work, experiments, acquisition, interpretation of data, drafting of work, final approval, agreement to be accountable for all aspects of work. SN: acquisition, interpretation of data, revising, final approval, agreement to be accountable for all aspects of work. RS: acquisition, revising, final approval, agreement to be accountable for all aspects of work. SD: acquisition, revising, final approval, agreement to be accountable for all aspects of work. ZC: interpretation of data, revising, final approval, agreement to be accountable for all aspects of work.

## Funding

This research was supported by a UC Davis funds and a Hellman Fellowship (AG).

### Conflict of interest statement

The authors declare that the research was conducted in the absence of any commercial or financial relationships that could be construed as a potential conflict of interest. The reviewer HSH and handling Editor declared their shared affiliation, and the handling Editor states that the process nevertheless met the standards of a fair and objective review.

## Abbreviations

FHC: familial hypertrophic cardiomyopathy
HcTnI: human cardiac troponin I
R204: arginine residue 204 on cardiac troponin I
TM: tropomyosin
Tn: troponin
TnC: troponin C
TnI: troponin I
TnT: troponin T
MOPS: 3-[N-morpholino]propanesulonic acid
WT: wild-type.
